# Trade-Off Between Triadimefon Sensitivity and Pathogenicity in a Selfed Sexual Population of *Puccinia striiformis* f. sp. *Tritici*

**DOI:** 10.3389/fmicb.2019.02729

**Published:** 2019-11-26

**Authors:** Yuan Tian, Yan Meng, Xiaocen Zhao, Xianming Chen, Hengbo Ma, Sanding Xu, Lili Huang, Zhensheng Kang, Gangming Zhan

**Affiliations:** ^1^State Key Laboratory of Crop Stress Biology for Arid Areas and College of Plant Protection, Northwest A&F University, Yangling, China; ^2^College of Pharmacy, Guizhou University of Traditional Chinese Medicine, Guiyang, China; ^3^Wheat Health, Genetics, and Quality Research Unit, United States Department of Agriculture-Agricultural Research Service, Washington State University, Pullman, WA, United States; ^4^Department of Plant Pathology, Washington State University, Pullman, WA, United States

**Keywords:** *Puccinia striiformis* f. sp. *tritici*, triadimefon, DMI resistance, *CYP51*, fitness

## Abstract

Stripe rust, caused by *Puccinia striiformis* f. sp. *tritici* (*Pst*), is one of the most destructive diseases of wheat and has been largely managed using demethylation inhibitor (DMI) fungicide triadimefon in China. To determine the sensitivity of *Pst*, a Chinese *Pst* isolate and its sexually produced progeny isolates were tested with triadimefon using the detached leaf method. The half maximal effective concentration (EC_50_) values varied greatly among the progeny isolates, ranging from 0.06 mg L^–1^ to 7.89 mg L^–1^. Twenty-six of the 56 tested progeny isolates were less sensitive to triadimefon than the parental isolate. A single-nucleotide mutation at the 401 position resulting in an amino acid change from tyrosine (Y) to phenylalanine (F) in the 134th codon (Y134F) of the cytochrome P450 sterol 14a-demethylase enzyme (*CYP51*), the target gene of DMI fungicide, was identified in the parental isolate. The 87 tested progeny isolates segregated into 19 homozygous wild type (AA), 40 heterozygous (AT), and 28 homozygous mutant (TT) genotypes, fitting a 1:2:1 ratio (χ^2^ = 2.43; *P* = 0.30). The mutant isolates had higher EC_50_ values than the wild type isolates. Significant differences in logEC_50_ were found between the mutant isolates and the wild type isolates (*P* = 2.2e^–16^). However, homozygous and heterozygous mutant isolates were not significantly different (*P* = 0.21), indicating dominant mutation. Twenty-two progeny isolates were used to inoculate a susceptible wheat variety, and latency period and lesion growth were recorded to compare wild type and mutant isolates for the pathogenicity fitness components. A moderate but significant negative correlation was detected between lesion growth and sensitivity to triadimefon (*r* = −0.53; *P* = 0.01). No significant variation in lesion growth was found between homozygous and heterozygous mutant isolates (*P* = 0.83). In the case of latency period and triadimefon sensitivity, no significant correlation was found (*P* = 0.17). These results are useful for understanding reduced sensitivity in the pathogen population and improving stripe rust management.

## Introduction

Stripe rust, caused by *Puccinia striiformis* Westend. f. sp. *tritici* Erikss. (*Pst*), is one of the most important diseases on wheat worldwide (Chen, 2005; Wellings, 2011). Growing resistant cultivars is an effective way to control stripe rust. However, new races of *Pst* can overcome race-specific resistance. Fungicides have been widely used to reduce stripe rust damage. Demethylation inhibitor (DMI) fungicides were introduced in agriculture in 1969 and have been widely used to control stripe rust (Line, 2002; Stammler et al., 2009; Kang et al., 2010). The use of DMI fungicides for decades have led to the emergence of strains with decreased sensitivity or even resistance in populations of various fungal pathogens, including *Blumeria graminis*, *Mycosphaerella graminicola*, and *Parastagonospora nodorum* (Wyand and Brown, 2005; Leroux et al., 2007; Pereira et al., 2017). *Pst* isolates less sensitive to DMI fungicides have been reported in the United Kingdom and the United States (Bayles et al., 2000; Kang et al., 2019). However, no fungicide insensitive isolates of *Pst* have been reported in China.

DMI fungicides interfere with the biosynthesis of the sterol in fungal membranes, ergosterol, by binding to the heme iron part of the cytochrome P450 sterol 14a-demethylase enzyme (CYP51) (Yuzo and Yuri, 1987). The CYP51 enzyme is widely distributed in various biological kingdoms, being found in animals, plants, fungi, and bacteria (Yoshida et al., 2000). Several fungi have been reported to contain multiple *CYP51* genes, for examples, *Fusarium graminearum* with 3 and different *Aspergillus* species have different numbers (*A. fumigatus* and *A. nidulans* with 2, and *A. orizae* with 3) of the CYR51 gene (Lepesheva and Waterman, 2007).

Three major molecular mechanisms have been found associated with resistance to azole compounds belonging to DMI fungicides in plant pathogenic fungi: (1) point mutations in the *CYP51* sequence, (2) overexpression of the *CYP51* enzyme, and (3) overexpression of genes encoding efflux pump proteins. Modification of the *CYP51* gene has been associated with altered triazole sensitivity in plant pathogenic fungi. Isolates of *Blumeria graminis* f. sp. *hordei* (*Bgh*) and *Blumeria graminis* f. sp. *tritici* (*Bgt*) with triadimefon resistance were detected in the fields with a point mutation in the *CYP51* gene, encoding a replacement of tyrosine for phenylalanine at position 136 (Y136F) (Délye et al., 1998). In addition, the combination of Y136F and K147Q were also identified in *Bgh* isolates with high resistance, while only K147Q in isolates were less resistant (Wyand and Brown, 2005). The Y144F and Y144H mutations in the *CYP51* gene of *P. nodorum* were correlated with significantly reduced sensitivity to the DMI fungicide propiconazole (Pereira et al., 2017). However, in *Puccinia triticina*, the wheat leaf rust pathogen, mutation Y134F was found at a low frequency and had no significant correlation to the epoxiconazole sensitivity. Overexpression of *CYP51* was also not the molecular mechanism for resistance to epoxiconazole (Stammler et al., 2009).

Sexual reproduction provides an effective method to study the genetic basis for various traits including fungicide sensitivity. In a cross between DMI-sensitive and resistant isolates of *Bgh*, both mutations segregate with resistance, which was consistent with *CYP51* controlling a major portion of DMI fungicide resistance (Robinson et al., 2002; Wyand and Brown, 2005). The previously developed *Pst* sexual populations (Tian et al., 2016, 2017) should be suitable for determining the genetic basis of fungicide sensitivity if the population segregates in this trait.

Fungicide resistance provides a selective advantage under fungicide selection, but resistance-conferring mutations may also result in fitness costs, resulting in an evolutionary trade-off (Hawkins and Fraaije, 2018). Fitness components of individual isolates between DMI-sensitive isolates and resistant isolates of *Cercospora beticola* showed that the resistant isolates had significantly lower virulence and spore production than the sensitive isolates, while differences in the other fitness components including incubation period, mycelial growth, germination of conidia, and germ tube length were insignificant (Karaoglanidis et al., 2001). Isolates of *Phakopsora pachyrhizi* with lower DMI sensitivity containing different *CYP51* or *CYTB* alleles had competitive disadvantages compared with wild-type *CYP51* isolates (Klosowski et al., 2016). However, no fitness costs were found in isolates of *Alternaria alternata* resistant to Quinone-outside inhibitor (QoI) fungicides or for *Phytophthora nicotianae* strains resistant to mefenoxam (Hu et al., 2008; Karaoglanidis et al., 2011).

In the present study, we used a *Pst* sexual population, which was developed by selfing isolate PL17-7 on barberry plants (Tian et al., 2017), to determine the relationships between fungicide sensitivity and fitness components. The specific objective were to (1) determine the triadimefon sensitivity levels of the parental isolate and the sexually produced progeny isolates and its inheritance; (2) determine the genotypes at the *CYP51* locus of the parental and progeny isolates, and compare the triadimefon fungicide sensitivity levels of isolates with the different *CYP51* genotypes; and (3) determine the relationships between triadimefon resistance and fitness components such as latency period and lesion growth.

## Materials and Methods

### Isolates and Multiplication

*Pst* isolate PL17-7 and its 87 progeny isolates previously developed through selfing the parental isolate on barberry plants (Tian et al., 2017) were used in the present study. The parental isolate PL17-7 was collected in 2013 from a wheat field in Gansu Province, where Triadimefon had been used to control stripe rust for many years. The development of the sexual population was previously described (Tian et al., 2016, 2017). Briefly, Leaves with teliospores were soaked in distilled water at room temperature for 24 h for germination according to Zhao et al. (2013). The petri dish containing wheat leaf segments with germinating teliospores was placed upside down on the top of the plastic cylinder surrounding a barberry seedling with two yong leaves, incubated in a dew chamber for 3–4 days in the darkness, and then grown in a growth chamber with a diurnal cycle of 12 h dark at 12°C and 12 h light at 16°C, 100% relatively humidity (RH). When nectars containing pycniospores appeared, about 7–8 days after inoculation, crosses were made by transferring nectar from one pycnium to another with toothpicks that had been soaked in sterile water for several hours. Aecia appeared on the opposite surface of the inoculated barberry leaves about 5–7 days after crossing. Individual aeciospores from aceial cups were transferred onto wheat leaves with a fine glass thread by the aid of a microscope. Isolates were multiplied on the seedlings of susceptible cultivar Mingxian 169 (MX169) (Zhang et al., 2001). Urediniospore suspension at the concentration of approximately 300,000 spores mL^–1^ in Novec 7100 (Mining and Manufacturing Company, Maplewood, MN, United States) was sprayed on 10-day-old seedlings (Sørensen et al., 2016). The inoculated plants were placed in a dew chamber at 10°C for 24 h in the darkness and transferred to the growth chamber at 16/10°C (day/night) with 16 h photoperiod. Urediniospores were collected using a clean glass tube 15 days after inoculation.

### Testing Fungicide Sensitivity

The parental isolate and 56 randomly selected progeny isolates out of the 87 progeny isolates were tested for sensitivity to triadimefon. Ten-day-old seedlings of MX169 were inoculated with urediniospores of each isolate collected the day before. Two microliter spore suspension in Novec 7100 (approximately 300,000 spores mL^–1^) was applied to the center of the first leaf of wheat with a pipette. The detached leaf method as described by Sørensen et al. (2016) was used to determine fungicide sensitivity. Triadimefon was added in water agar (0.6% agar, 60 mg L^–1^ 6-Benzylaminopurine) with various concentrations in different petri dishes. Seven days after *Pst* inoculation, three-centimeter leaf segments were cut from the inoculated leaves and placed in petri dishes on water agar with different concentrations of triadimefon (12.0, 6.0, 3.0, 1.5, 0.8, 0.4, 0.2, 0.1, and 0.05 mg L^–1^). Water agar with only 6-Benzylaminopurine at the concentration of 60 mg L^–1^ was used as a non-triadimefon control. The petri dishes with detached leaves were kept in a growth chamber at 16/10°C (day/night) with 16 h light. Digital images were taken 15 days post inoculation. Lesion length was measured with a ruler. For each treatment, five leaf segments were used in the measurement. The experiment was conducted twice. The EC_50_ value, which is the effective concentration of active ingredient to inhibit 50% lesion growth, was determined for each isolate using the lesion lengths at different triadimefon concentrations. An isolate of race CYR29 that was collected more than 10 years ago and found to have the lowest EC_50_ value of 0.15 mg L^–1^ among 80 isolates, which were collected from wheat fields over 10 years and tested in a preliminary experiment (data not shown), was used as a reference in the present study. The resistance factor was determined for each isolate by dividing its EC_50_ by that of the reference isolate. Isolates with resistance factor values higher than 3.92 were considered resistant to triadimefon and isolates lower than 3.92 were treated as sensitive.

### Identification of the *CYP51* Gene in *Pst*

The *CYP* gene family of *Pst* was identified using software HMMER 3.0 (*e* ≤ 10^–10^) with the hmmsearch of profile hidden Markov model derived from the Pfam seed alignment flatfile of PF00067 against the *Pst* proteome (unpublished). Domain analysis was performed using software Pfam 31.0 for all matching sequences. Thirty-seven protein sequences of the *CYP51* gene belonging to 26 species of fungi from phyla Ascomycota, Basidiomycota, Chytridiomycota, and Zygomycota were used in this study (Table 1). The protein sequences of *CYP51* genes in different species were downloaded from the cytochrome P450 webpage of fungi^1^, Fungal cytochrome P450 database^2^, or BLAST search against the NCBI database by blastp using the *CYP51* protein sequence of *Puccinia triticina* as a query (gene accession number: FJ976683) (Stammler et al., 2009). The *CYP51* protein sequence of bacterium *Streptomyces coelicolor* was used as an outgroup. The related information is presented in Table 1.

**TABLE 1:** Fungal species used to compare sequences of gene *CYP51*.

**Fungal species**	**Phylum**	**Class**	**Number of *CYP51* genes**	**Database**
*Mycosphaerella fijiensis*	Ascomycota	Dothideomycetes	1	Fungal cytochrome P450
*Magnaporthe oryzae*	Ascomycota	Sordariomycetes	2	Fungal cytochrome P450
*Neurospora crassa*	Ascomycota	Sordariomycetes	1	Fungal cytochrome P450
*Sclerotinia sclerotiorum*	Ascomycota	Leotiomycetes	1	Fungal cytochrome P450
*Botrytis cinerea*	Ascomycota	Leotiomycetes	1	Fungal cytochrome P450
*Aspergillus terreus*	Ascomycota	Eurotiomycetes	3	Fungal cytochrome P450
*Aspergillus flavus*	Ascomycota	Eurotiomycetes	3	Fungal cytochrome P450
*Neosartorya fischeri*	Ascomycota	Eurotiomycetes	2	Fungal cytochrome P450
*Coccidioides immitis*	Ascomycota	Eurotiomycetes	2	Fungal cytochrome P450
*Yarrowia lipolytica*	Ascomycota	Saccharomycetes	1	Fungal cytochrome P450
*Saccharomyces kluyveri*	Ascomycota	Saccharomycetes	1	Fungal cytochrome P450
*Neurospora discreta*	Ascomycota	Saccharomycetes	1	Fungal cytochrome P450
*Chaetomium globosum*	Ascomycota	Saccharomycetes	1	Fungal cytochrome P450
*Fusarium oxysporum* f. sp. *lycopersici*	Ascomycota	Saccharomycetes	3	Cytochrome P450 webpage of fungi
*Fusarium verticillioides*	Ascomycota	Saccharomycetes	3	Cytochrome P450 webpage of fungi
*Ustilago maydis*	Basidiomycota	Ustilaginomycetes	1	Fungal cytochrome P450
*Melampsora larici-populina*	Basidiomycota	Urediniomycetes	1	Fungal cytochrome P450
*Puccinia graminis* f. sp. *tritici*	Basidiomycota	Urediniomycetes	1	NCBI
*Puccinia horiana*	Basidiomycota	Urediniomycetes	1	NCBI
*Puccinia triticina*	Basidiomycota	Urediniomycetes	1	NCBI
*Phanerochaete chrysosporium*	Basidiomycota	Agaricomycetes	1	Fungal cytochrome P450
*Malassezia globosa*	Basidiomycota	Exobasidiomycetes	1	Fungal cytochrome P450
*Cryptococcus neoformans* var. *neoformans*	Basidiomycota	Tremellomycetes	1	Fungal cytochrome P450
*Sporobolomyces roseus*	Basidiomycota	Microbotryomycetes	1	Fungal cytochrome P450
*Batrachochytrium dendrobatidis*	Chytridiomycota	Chytridiomycetes	1	Fungal cytochrome P450
*Phycomyces blakesleeanus*	Zygomycota	Chytridiomycetes	1	Fungal cytochrome P450
*Streptomyces coelicolor*^a^	Actinobacteria	Actinobacteria	1	Fungal cytochrome P450

*^*a*^Used as an out group.*

All protein sequences were aligned in Clustal Omega v. 1.2.4 with default parameters and the alignments were further trimmed in Trimal v. 1.2 using a gap threshold of 0.25 (Capella-Gutiérrez et al., 2009; Sievers and Higgins, 2014). A phylogenetic tree was constructed using maximum-likelihood (ML) in IQTREE v1.6.1 with ultrafast bootstrap value (UFBoot) set to 1000 and the protein model set as LG + R6 estimated using IQTREE (Nguyen et al., 2014).

### Sequence Variation in the *CYP51* Gene

DNA was extracted from urediniospores of the parental isolate and the 87 progeny isolates using the cetyltrimethylammonium bromide (CTAB) method (Chen and Ronald, 1999). DNA integrity was checked using 1.2% agarose gel electrophoresis, and the concentration was determined using NanoDropTM 1000 (Thermo Fisher Scientific, United States). The parental isolate PL17-7 and 60 progeny isolates selected based on their different genotypes using 13 SSR markers for screening were sequenced using the Illumina sequencing technology (Tian et al., 2016). High-quality reads were mapped to the reference sequence of *Pst*-CY32 using software Burrows–Wheeler Aligner (BWA), and SNPs and Indels were identified using the Genome Analysis Toolkit (GATK) v3.3.0 (DePristo et al., 2011) and corrected manually. The mutants in the *CYP51* gene in the parental isolate and 87 progeny isolates were confirmed by genotyping using Kompetitive Allele Specific PCR (KASP) primers KaspF1 (5′-ga aggtgaccaagttcatgctCTGTATTCGGTACGGATGTAGTTTA-3′), KaspF2 (5′-gaaggtcggagtcaacggattCTGTATTCGGTACGGATGT AGTTTT-3′), and KaspR (5′-AAGATTGCGTTCGGGACAT-3′), in which the detective primer sequences of FAM (gaaggtgaccaagttcatgct) and HEX (gaaggtcggagtcaacggatt) were added to the 5′ of forward primers. These KASP primers were used to detect the mutant specifically at the 401th nucleotide position. Reaction mixtures in the final volumes of 5 μL containing 2.5 μL of genomic DNA (50–100 ng), 2.5 μL of 2 × KASP master mix (V4.0, LGC Genomics), and 0.056 μL of primer mix (12 μM of each allele-specific primer and 36 μM of common primer). Polymerase chain reaction (PCR) was performed under cycling conditions: denaturation at 94°C for 15 min, 10 cycles of 94°C for 20 s, touchdown starting at 65°C for 60 s (decreasing 0.8°C per cycle), and followed by 32 cycles of amplification (94°C for 20 s; 57°C for 60 s). End-point fluorescence was visualized with a microplate reader (FLUOstar Omega, BMG LABTECH, Germany) and analyzed using software Klustering Caller (LGC, Middlesex, United Kingdom).

### Pathogenicity Fitness Analysis

Latency period and lesion growth were used as pathogenicity fitness components to evaluate 22 progeny isolates, including 4 with the wild type (AA), 9 with the heterozygous mutant genotype (AT), and 9 with the homozygous mutant genotype (TT). The method described by Sørensen et al. (2016) was used to measure the two components with modifications. Ten MX169 wheat plants per isolate were inoculated with one microliter urediniospore suspension (approximately 300,000 spores mL^–1^) diluted by Novec 7100 onto the center of first leaves with a pipette. Seven days after inoculation, the plants were examined every 12 h in order to determine the time period between inoculation and appearance of the first lesion, which was considered as the latency period and recorded when the first lesion appeared on more than half of the ten plants in each replication. Two days after the appearance of first lesions, the length of lesions was measured using a ruler and recorded again 6 days after the appearance of first lesions. The lesion growth rate per day was obtained by dividing the difference of lesion lengths between the two recordings in a 4-day interval by 4. The test was repeated twice using a complete randomization design in each replication.

### Data Analyses

Fungicide sensitivities determined by EC_50_ were estimated for the parental isolate and 56 progeny isolates. The EC_50_ values were determined by linear regression of probit-transformed relative inhibition value against the log_10_ of fungicide concentration using software SPSS (Statistical Product and Service Solutions, version 23.0, Armonk, NY, United States). The significant difference in sensitivity to triadimefon in the term of logEC_50_ value of sensitive isolates (similar to the reference isolate and also with the AA *CYP51* genotype) and resistant isolates (significantly different from the reference isolate and with the TT or AT genotype at the CYP51 locus), and the difference between the homozygous and heterozygous point mutations was assessed using the Student’s *t-*test. Fitness parameters were also compared using the Student’s *t-*test. Correlation coefficients between the fitness components and sensitivity to triadimefon in the term of logEC_50_ value were estimated. All these statistical analyses were performed using R version 3.4.0.

## Results

### Sensitivity of *Pst* to Triadimefon

**FIGURE 1:**
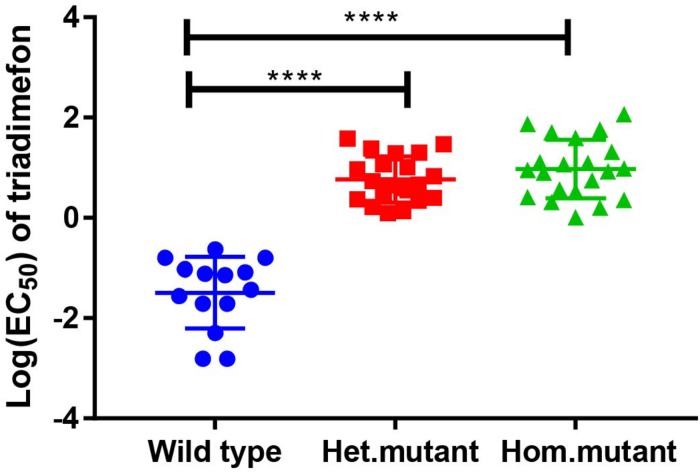
Responses of the parental isolate and progeny isolates to triadimefon (estimated as EC_50_) and *t*-test of different genotypes (^****^P < 0.001).

Variation in triadimefon sensitivity was observed among the tested 56 progeny isolates as indicated by a wide range of EC_50_ values, from 0.06 mg L^–1^ to 7.89 mg L^–1^ (Figure 1 and Table 2). The EC_50_ of the most resistant isolate was more than 131 times as the most sensitive one among the tested progeny isolates. The parental isolate had an EC_50_ value of 1.88 mg L^–1^ (Table 2). Fourteen progeny isolates were sensitive to triadimefon, while the others had different levels of insensitivity. Twenty-six of the progeny isolates were more resistant than the parental isolate (Table 2).

**TABLE 2:** Nucleotide genotype of the *CYP51* gene at the 401 position of *Puccinia striiformis* f. sp. *tritici* (*Pst*) isolates and their EC_50_ value, sensitivity reduction factor, latency period, and lesion growth.

***Pst* isolate**	**CYP51 genotype^a^**	**EC_50_ (mg/L)**	**Resistance factor**	**Latency period (hour)**	**Lesion growth (cm)**
11	AA	0.54	3.59	228	0.62
32	AA	0.10	0.67	222	0.57
39	AA	0.45	3.00	216	0.62
82	AA	0.45	2.98	228	0.68
76	AA	0.32	2.13	–^c^	–
a1	AA	0.18	1.18	–	–
a10	AA	0.06	0.40	–	–
a11	AA	0.18	1.17	–	–
a17	AA	0.21	1.37	–	–
a18	AA	0.24	1.60	–	–
a23	AA	0.33	2.19	–	–
a24	AA	0.36	2.37	–	–
a4	AA	0.34	2.27	–	–
a9	AA	0.06	0.37	–	–
a12	AA	–	–	–	–
a2	AA	–	–	–	–
a26	AA	–	–	–	–
a27	AA	–	–	–	–
a28	AA	–	–	–	–

	**Mean**	**0.27**	**1.81**	**223.50**	**0.62**

4	AT	1.46	9.74	228	0.56
23	AT	1.24	8.27	228	0.37
42	AT	3.67	24.47	240	0.40
45	AT	1.42	9.46	216	0.53
52	AT	3.62	24.13	228	0.56
66	AT	2.31	15.40	232	0.41
72	AT	2.74	18.27	222	0.54
113	AT	1.80	11.98	216	0.51
116	AT	2.65	17.67	216	0.52
16	AT	2.08	13.87	–	–
19	AT	4.35	29.00	–	–
24	AT	4.87	32.47	–	–
49	AT	1.95	13.00	–	–
55	AT	2.92	19.47	–	–
56	AT	1.91	12.73	–	–
79	AT	4.02	26.80	–	–
a19	AT	1.11	7.39	–	–
a20	AT	1.49	9.95	–	–
a21	AT	1.74	11.58	–	–
a3	AT	1.15	7.69	–	–
a5	AT	1.62	10.82	–	–
PL17-7^b^	AT	1.88	12.53	–	–
3	AT	–	–	–	–
7	AT	–	–	–	–
10	AT	–	–	–	–
21	AT	–	–	–	–
27	AT	–	–	–	–
31	AT	–	–	–	–
33	AT	–	–	–	–
35	AT	–	–	–	–
51	AT	–	–	–	–
53	AT	–	–	–	–
59	AT	–	–	–	–
61	AT	–	–	–	–
65	AT	–	–	–	–
68	AT	–	–	–	–
73	AT	–	–	–	–
77	AT	–	–	–	–
89	AT	–	–	–	–
110	AT	–	–	–	–
a7	AT	–	–	–	–

	**Mean**	**2.36**	**15.76**	**225.11**	**0.49**

2	TT	2.09	13.93	240	0.4
30	TT	7.89	52.60	222	0.47
37	TT	3.02	20.13	234	0.42
40	TT	1.44	9.57	228	0.54
41	TT	2.90	19.33	228	0.49
63	TT	6.52	43.47	232	0.48
78	TT	2.96	19.73	234	0.47
81	TT	1.38	9.20	236	0.54
118	TT	5.49	36.60	234	0.53
1	TT	5.81	38.71	–	–
22	TT	2.67	17.80	–	–
36	TT	4.94	32.93	–	–
54	TT	3.75	25.00	–	–
85	TT	2.59	17.27	–	–
a8	TT	1.01	6.75	–	–
a13	TT	1.70	11.36	–	–
a14	TT	1.50	10.01	–	–
a16	TT	2.53	16.88	–	–
a22	TT	1.77	11.78	–	–
a25	TT	2.45	16.34	–	–
a29	TT	1.22	8.11	–	–
17	TT	–	–	–	–
25	TT	–	–	–	–
104	TT	–	–	–	–
108	TT	–	–	–	–
112	TT	–	–	–	–
117	TT	–	–	–	–
a15	TT	–	–	–	–

	**Mean**	**3.13**	**20.83**	**232.00**	**0.48**

*^*a*^AA = wild type [nucleotide AA at the 401 position; amino acid tyrasine (Y) at the 134 position]; TT = homozygous mutant [nucleotide TT at the 401 position; amino acid phenylalanine (F) at the 134 position]; and AT = heterozygous (dikaryotic) genotype. ^*b*^The parental isolate. ^*c*^Not tested.*

### Identification of the *CYP51* Gene of *Pst*

**FIGURE 2:**
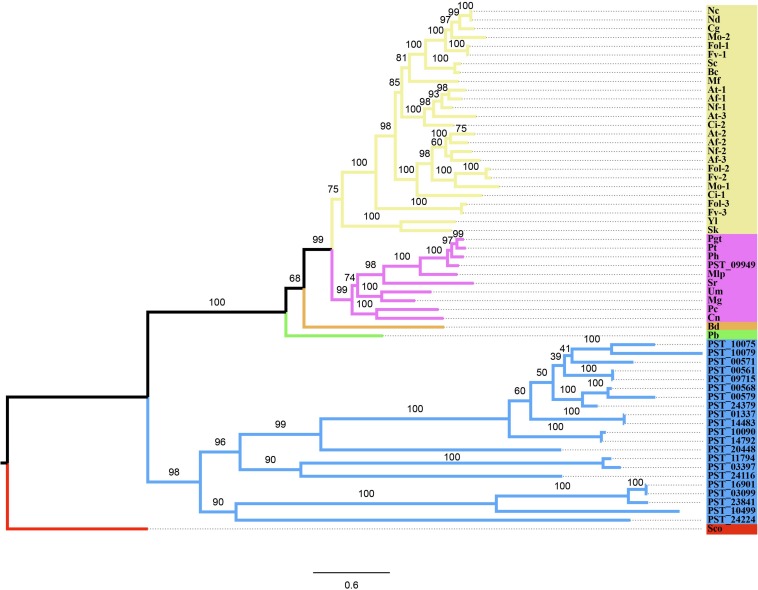
Phylogenetic tree based on the *CYP51* gene amino acid sequences of 26 fungal species in different phyla and predicted 22 genes in the CYP family of *Puccinia striiformis* f. sp. *tritici* (*Pst*) Spokes highlighted in blue present the CYP family of *Pst*; spokes in green are Zygomycota; spokes in orange Chytridiomycota; spokes in purple Basidiomycota; spokes in yellow Ascomycota; and the outgroup *Streptomyces coelicolor* (Sco) is in red. The numbers around branch nodes were ultrafast bootstrap support. The corresponding relation for full names and abbreviations are showed as follows: Nc, *Neurospora crassa*; Nd, *Neurospora discreta*; Cg, *Chaetomium globosum*; Mo, *Magnaporthe oryzae*; Fol, *Fusarium oxysporum* f. sp. *lycopersici*; Fv, *Fusarium verticillioides*; Sc, *Sclerotinia sclerotiorum*; Bc, *Botrytis cinerea*; Mf, *Mycosphaerella fijiensis*; At, *Aspergillus terreus*; Af, *Aspergillus flavus*; Nf, *Neosartorya fischeri*; Ci, *Coccidioides immitis*; Yl, *Yarrowia lipolytica*; Sk, *Saccharomyces kluyveri*; Pgt, *Puccinia graminis* f. sp. *tritici*; Pt, *Puccinia triticina*; Ph, *Puccinia horiana*; Mlp, *Melampsora larici-populina*; Sr, *Sporobolomyces roseus*; Um, *Ustilago maydis*; Mg, *Malassezia globosa*; Pc, *Phanerochaete chrysosporium*; Cn, *Cryptococcus neoformans* var. *Neoformans*; Bd, *Batrachochytrium dendrobatidis*; Pb, *Phycomyces blakesleeanus.*

Twenty-two genes of CYP family of *Pst* were identified using software HMMER against the *Pst* proteome (Supplementary Table 1). The PF00067.21 domain was identified in all of the 22 genes using software Pfam. The phylogenetic tree analysis of the *Pst* CYP family and CYP51 proteins of 26 fungi showed that these proteins were clearly divided into two clades. *PST_09949* was the only *CYP51* gene in *Pst*. This gene encoding 545 amino acids was in the group with *Puccinia graminis* f. sp. *tritici*, *Puccina horiana*, *P. triticina*, and *Melampsora larici-populina*, all rust fungi (Figure 2).

### Analysis of the *CYP51* Sequences of *Pst* Isolates

The complete sequences of *CYP51* in the parental isolate and 60 progeny isolates were analyzed to identify nucleotide variation. Only one non-synonymous substitution, changing from A to T at the 401th nucleotide position resulting in the change from tyrosine (Y) to phenylalanine (F) at codon position 134 (Y134F), was found in *CYP51* of parental isolate PL17-7 and all resistant progeny isolates, but absent in the sensitive progeny isolates. The presence and absence of the mutation was confirmed using the KASP marker in the parental isolate and all progeny isolates.

### Correlation Between Fungicide Resistance and Sequence Variation

The parental isolate was heterozygous at the mutant locus. The 87 progeny isolates segregated at this locus with the homozygous wild type (AA), heterozygous mutant (AT), and homozygous mutant (TT) genotypes. The EC_50_ values of the isolates with the homozygous wild type genotype varied from 0.06 to 0.54 mg L^–1^, with a mean value of 0.27 mg L^–1^, those of the isolates with the heterozygous mutant genotype ranged from 1.11 to 4.87 mg L^–1^ with the mean of 2.36, whereas the values of the isolates with homozygous mutant genotype ranged from 1.01 to 7.89 mg L^–1^ with the mean of 3.13 mg L^–1^ (Table 2). There was a significant difference in logEC_50_ between the mutant isolates including both heterozygous and homozygous mutant genotypes and the wild type isolates (*P* = 2.2e^–16^) (Figure 1). The difference between the heterozygous mutant and the wild type isolates was significant, and so was the difference between the homozygous mutant and wild type isolates (Figure 1). However, the difference in logEC_50_ between the homozygous and the heterozygous mutant isolates was not significant (*P* = 0.21). The segregation of wild type, heterozygous mutant, and homozygous mutant genotypes fitted a 1:2:1 ratio (χ^2^ = 2.43; *P* = 0.30).

### Pathogenicity Fitness

**FIGURE 3:**
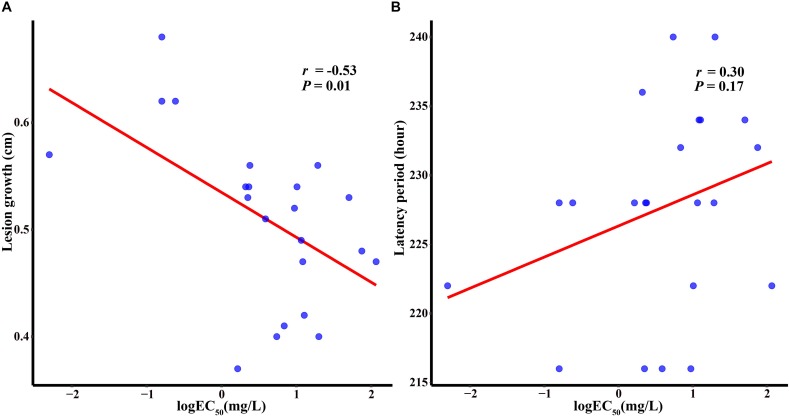
Correlations between the level of sensitivity to triadimefon and length growth **(A)** and latency period **(B)**.

Two components were measured for 22 isolates including four sensitive isolates and 18 sensitivity-reduced isolates (nine isolates with the heterozygous mutant genotype and nine isolates with the homozygous mutant genotype). Variation was found among these isolates for both fitness components. In the sensitivity-reduced isolates, the lesion growth ranged from 0.37 to 0.56 cm per day, with a mean of 0.49 cm, whereas in the group of sensitive isolates (the wild type genotype), the lesion growth ranged between 0.57 and 0.68 cm, with a mean of 0.62 cm per day. Sensitivity-reduced isolates and sensitive isolates had a significant difference in lesion growth (*P* = 0.0005). Isolates with the homozygous mutant genotype and those with the heterozygous mutant genotype showed no significant difference in lesion growth (*P* = 0.83). For latency period, sensitivity-reduced isolates had the values ranging from 216 h to 240 h, with a mean of 228.56 h, while sensitive isolates had the values ranging between 216 and 228 h, with a mean of 223.5 h. Though sensitivity-reduced isolates had a high value than sensitive isolates, the difference was not significantly different (*P* = 0.23). The correlation coefficients of the two fitness components and sensitivity to triadimefon were determined by the logEC_50_ values. The lower lesion growth was, the more reduced sensitivity to triadimefon (*r* = −0.53; *P* = 0.01). However, no significant correlation was found between latency period and triadimefon sensitivity among the genotype groups (*P* > 0.05) (Figure 3).

## Discussion

The use of Azole fungicides for decades has led to the emergence of strains with reduced sensitivity or even resistance in various plant fungal pathogens. Isolates of both barley (*Bgh*) and wheat (*Bgt*) powdery mildew pathogens with highly resistance to triadimefon have been identified in the field (Wyand and Brown, 2005). In China, the DMI fungicide triadimefon, an azole chemical, has been used since the late 1970s for controlling wheat stripe rust and other plant diseases. In our study, the parental isolate obtained from Gansu Province in 2017 was found with reduced sensitivity to triadimefon (EC_50_ = 1.88 mg L^–1^) compared with many other isolates collected from various provinces (data not shown). This was the first report about reduced sensitivity to triadimefon in the *Pst* population in China.

Rust fungi produce polycyclic, abundant air-borne urediniospores. It would be wise to vigilantly monitor the emergence of resistance in rust fungi (Oliver, 2014). More isolates should be collected from various regions and tested for *CYP51* mutants in order to monitor the distribution and spreading of triadimefon-resistant isolates. To prevent selection of insensitive isolates in the field, other fungicides with different modes of action should be used in mixture or rotation for control of stripe rust.

*CYP51* is a sterol 14α-demethylase belonging to the cytochrome P450 monooxygenase (CYP) superfamily. The enzyme plays an important role in the biosynthesis of ergosterol which is a sterol specifically found in fungal membranes for mediating membrane permeability and fluidity (Daum et al., 1998). Mutations in the *CYP51* gene of *B. graminis*, *M. graminicola*, and *P. nodorum* at one or more nucleotide positions were found to be associated with resistance to DMI fungicides (Wyand and Brown, 2005; Leroux et al., 2007; Pereira et al., 2017). In the present study, the progeny isolates were separated into the sensitive group (EC_50_ from 0.06 to 0.54 mg L^–1^) and sensitivity-reduced group (EC_50_ range from 1.01 to 7.89 mg L^–1^). All progeny isolates in the reduced sensitive group had either one copy (heterozygous) or two copies (homozygous) of the Y134F substitution, while the sensitive isolates (homozygous wild-type) did not have the substitution mutation. The difference of logEC_50_ between the mutant isolates and wild-type isolates was significant (*P* = 2.2e^–16^), indicating that the mutation in the *CYP51* gene was associated with the reduced sensitivity to triadimefon. This funding was different from a report that a *CYP51* mutant in *P. triticina* did not confer significant changes in the sensitivity to triazole (Stammler et al., 2009), but consistent with many reports on resistance to this type of fungicides in other fungal pathogens (Wyand and Brown, 2005; Leroux et al., 2007; Pereira et al., 2017).

Sexual reproduction, when it occurs, plays an important role in producing new recombinant genotypes in fungal populations (De Miccolis Angelini et al., 2015). Sexual reproduction of *Pst* under natural conditions has been found in China (Zhao et al., 2013; Wang et al., 2016). Under controlled conditions, sexual reproduction has been demonstrated to producing diverse progeny isolates (Tian et al., 2016, 2017; Wang et al., 2018; Yuan et al., 2018; Siyoum et al., 2019). Our results also demonstrated that recombinant genotypes could be obtained through sexual reproduction. Isolates developed from selfing on barberry had different levels of sensitivity to triadimefon and some of the progeny isolates especially of the homozygous mutant genotype had higher EC_50_ values than the heterozygous parental isolate. If occur, sexual reproduction in the field may also accelerate the development of less sensitive or resistant isolates.

There is a concept that resistance to pesticides of non-haploid organisms would be recessive. However, in the study of *Rhizoctonia cerealis*, thifluzamide resistance was found to be dominant as heterozygous TR mutants possess the same phenotype of the homozygous mutants (Mu et al., 2014). Our result was in consistent with this study. The progeny isolates were segregated into homozygous mutants, heterozygous mutants, and homozygous wild type, at a 1:2:1 ratio. This result showed that sensitivity to triadimefon was controlled by a single gene, *CYP51*, and the reduced sensitive phenotype was dominant. Thus, only one step mutant can make a homozygous wild type isolate become less sensitive. In the present study, we found only one nucleotide mutation within *CYP51* by sequencing. However, if two or more mutation sites occur within the gene, the pathogen may become more resistant (Wyand and Brown, 2005; Leroux et al., 2007). In order to know if there are other mutant sites in *Pst*, more isolates collected from fields should be studied.

Fungicide resistance may reduce pathogen fitness. There is an evolutionary trade-off between fungicide resistance and the cost of fitness. Resistant isolates may not be prevalent if fitness costs exist. Such fitness costs should be taken into account when we assess the resistance risk (Hollomon, 2016). Resistance mutations can disrupt or reduce the efficiency of important physiological and biochemical process in the pathogen, leading to lower fitness (Klosowski et al., 2016). Allelic variants may cause different levels of resistance and/or different negative pleiotropic effects on the fitness of resistant mutants (De Miccolis Angelini et al., 2015). Cases of lower competitive ability of resistant isolates than that of the sensitive isolates have been reported in many fungal populations. The measurements of fitness components of *C. beticola* individual isolates showed that the resistant isolates had significantly lower virulence and spore production than the sensitive isolates, while no significant differences to fitness components of mycelial growth, spore germination, germ tube length, and incubation period (Karaoglanidis et al., 2001). In *A. alternate*, no significant differences in sporulation *in vitro*, mycelial growth, incubation period, and sporulation *in vivo* were found between QoI fungicide resistant and sensitive isolates (Karaoglanidis et al., 2011). In *P. pachyrhizi*, the soybean rust pathogen, isolates with lower DMI sensitivity had competitive disadvantages compared with those of sensitive isolates (Klosowski et al., 2016). In the present study, two pathogenicity-related fitness parameters of isolates were measured. A negative correlation was found between fungicide insensitivity and lesion growth (*r* = −0.53; *P* = 0.01). Less sensitive isolates developed at slower rates than sensitive isolates when lesion growth was measured. However, no clear correlation was found between latency period and fungicide sensitivity. The results indicated that the *CYP51* mutation did not significantly influence the latency period. Isolates with heterozygous mutant and isolates with homozygous mutant showed no significant difference on lesion growth and latency period. For obligate biotrophic fungi like *Pst*, some parameters, such as latency period and lesion length, that are related pathogenicity are also related to fitness. Fungicide insensitive isolates have the selection advantage under fungicide selection, but are disadvantageous under non-fungicide selection, as demonstrated in the present study. Further studies are needed to determine if fitness cost exists for reduced fungicide sensitivity by testing other parameters of fitness or aggressiveness, especially survival, under various environmental conditions. Nevertheless, the current study provides the basic EC_50_ values of the wild-type and mutant isolates for continual monitoring the *Pst* population related to fungicide sensitivity.

## Data Availability Statement

This manuscript contains previously unpublished data. The name of the repository and accession number(s) are not available.

## Author Contributions

YT, YM, XZ, HM, and SX participated in isolates collection, fungicide sensitivity testing, DNA extraction, SNP genotype analyses, and fitness analyses. YT, YM, and HM analyzed the data. YT wrote and edited the manuscript. XC revised the manuscript. LH, ZK, and GZ conceived the study and designed the experiments. All authors reviewed and approved the final version of the manuscript.

## Conflict of Interest

The authors declare that the research was conducted in the absence of any commercial or financial relationships that could be construed as a potential conflict of interest.

## Footnotes

1 http://drnelson.uthsc.edu/fungal.genomes.html

2 http://p450.riceblast.snu.ac.kr/index.php?a=view
