# Supplementary material for: Trade-Off Between Triadimefon Sensitivity and Pathogenicity in a Selfed Sexual Population of Puccinia striiformis f. sp. Tritici
Source: Front Microbiol. 2019 Nov 26;10:2729. doi: 10.3389/fmicb.2019.02729 (PMC6901989; doi:10.3389/fmicb.2019.02729)
Supplement: Supplementary file 1 [file Table_1.DOCX]

**Supplement Table 1.** CYP family genes identified from the *Puccinia striiformis* f. sp. *tritici* CY32 proteome

| Accession no. | Amino acid sequence |
| --- | --- |
| PST_00561 | MTSRNRAIGTTKRKDPAFHEDPGWPLTNLYRAQRVGQLPTVIKNRARSLEEMTMRGLKYGPGHAITVPGVRMIDVSKPEWLEYIQKTNFDNYVKGPLFRNCMYDVFGDGIFVSDGQMWKRARHATSTIFTLKTFKTIIVPSSNKSMDELSQVLTSIAGRNHPIDFCDLFFRFTLDSFVHMTFGKDLGLLGIGYEDKGSNPFSVAFDYAQDQVDFRFVMPIGWRLIERLNIRSMGKRMKVSCGVLDDYAYSLIDERMSNLTSDPKKEKQATIHTDLLGLFMNARDERGGDLGRTELRDTTLNLIIAEFAFSDTTAEVLSWSFFHLLMNEDLVLRIREEASGILGEEKRVTYENYKQFILAYAIVHETLRLHPSVPKDLKCVASDDQIPGGPRVEAGDMEGANFLYLYLWVVSHNQSDWQMGRDASIWGPDCGEFKPDRWIDDEKGSIKQFGPYKFHAFNAGPRICLGMNLAIYQAVKVIVETFMSFELEFAPGWLENVPKSESIEGITSRYPTPMYRTSLTLPMDNPMMISVKKKKLPLVDM |
| PST_00568 | MIANRSRFLEGITRIALKFGPGFSFTLPGKRMIDVSKPEWVEYIQKTNFNNYVKGPMIYPAMSDVWGSIIIAVDGPAWKRTRQAIVSIFAPKTFKTIIVPSMNQSMIGFAQVLTTAAEERLPIDICDLFLRFTLDSFVRMTFSKDLGICDARYLNHSSSISAAPEPSDSTSRFAEALDFSQSQVDFRLSIMIGWKLIEWLNIGSMGKRMKESCQVLDEFIYPLIDKRLADSSRKPDLDAKESSHPDLLSLFITTRDERGGGLGRTELKEAALTLIIAGRDTTAQSLSWAFFHLLMNKDLISKIREETIEVLAVVLEALRLHPGVPKNVKLAVNADKIPDGPTIEAGDIVRWSDWQMARDPSIWGDDCGEFKPQRWFDDTGALRQFGQFKFHAFNGGPRLCPGMNLAILEAVKVIVQVLRDFELEFAEGWLENVPKSELIEGVASRYRTPTYGSSISLPMENPMMISVRLRQLD |
| PST_00571 | MEGLTDELRSAAGEEHSIDVCDLFFRFTLNSVAQMTFGKDLDLLGAKYSAETDSPASSKLAHSVNEFVDAFDFAQEQSNFRLTLIVGWKLIESMNSSMGKRMKHSIRIIDNFVYFLLDERIASLSRPDRLKEAEHVPKDLLGLFMDARDERGGGLSRTELRDTAVGLIFGARDSTAQTMSWALFHLLMNKDLVTRVREEAAKVLGDQQGDRSKVTYANHKQFVVAHAIILETLRLHPSAPKVTCYSMSLEVLNAELLSICQRYQYSRLPQSAKIALADDKIPDGPTIHAGDVLRWSDWQIGRDVTVWGPDCGEFKPDRWFDETGRIKQFGQFKFHAFNGGPRLCLGMSLAKFEAIKIMVEVLQNFDLEFADGWLENVPKTEAIEGVPSRYPVPAYKSSVTFPMRHPMMVIVKPHAN |
| PST_00579 | MDVSKPEWVEYIQKKLVSSHQQFRLRLLFSHDRSLHPNHSPMVFFVANFNNYIKGPMIYSAMPDVWGRIIISADGPAWKRTRQAMVSIFTPKTFKVDDPESTLILYSKIHYYTHLISKIREETIEVLGDDGVDDQQSVTYENYKQFLWAQAVVLEALRLHPGVPKNVKLAVNADKIPGGPTIEAGDIVRWSDWQMARDPSIWGDDCGNSNHSGGSMIQTKGSTIFLTCNHYQNLPHILKTNPIFFTSHLFYGGPRHCPGMNLSILEAVKVIVQVLRDFELEFAEGWLENVPKSEFIEGVASQYGTPTYGSSISLPMENPMMISVRLRRRD |
| PST_01337 | ILGQIPEIIMNFSRPLELGMARHLKFRPGWSVTMPGLRLIDISKPEWLEYVQKTNFHNYVKGPRFHSVMADVFGNGIFVTDGAQWKKSRHLLAPLFTIKTFKVCISPALRTNLDTLHESMQLASESRPVIDICDVLFKFSMNFLVYATLGKDMGQLDQLHLPSIQPSSPSADSKFVDAFEHAQKQLDLRFALLTSWKVVEKVNVSMGRKIASACQTLHDYASLLIDERLTSLGQGPQEYDETPTDLLGFFIKNRKEMGGDLDQAELKETFLNLIIAGRDSTAEALTWAFYHLLMNKDIVNKIREEASEVTGKDRSGQVTYENSKEFKWAHAVILEALRLHPSIPKNVRFALKADKIPGGPVIEAGDAVRWSDWAMARDPQVWGDDCLEFRPARWIDDNGNIKQFGHFKFHAFNGGPRVCLGMNFAILQCICMIVEVFHNFELELEPEWLAQVPKTPIVGHTSPNAYGTPKYKPSLTLPMAQSMMVTIKPY |
| PST_03099 | MTELIPATMRLIHNEIVQNFRVIFSLFICLLSPVILSTITRRRRCLPLPPGPKSYPFMRYSFGPYQWRQMEEITKEYGPVSSVKLGNNKLLVIVGRVEPALALLEGRSSIYCDRPQLEMAGNIMSGGLRTLCLPYGDRWKRFRRVLHSQLDTKAATSYQSIQERASRQMILDIVERPKDFTEALTRYAASVIIKITYGKLTPIHHGDAEVVKVIKTLSRFTRATRIGAHSVDRFPWLRHIPGWVAQGRKWHQEELDLFSSQVDGVRSEMNIPGKRDSCFTSYMLERQKEFSLSDNEAAYLAGSLFGAGSDTTSAALAVVIFAAASHPSEVKKVQDELESVVGNGRMPTFD  DYPELPWVSAFVSETFRWRPVSAAGFMHATVKDDVYEGHFIPAGSWVIGNHWSIHRDESVFPEPEKFDLTRWLTIDEESGKTVLRPDMRHFAYGFGRRRCAGVTIADRSMFINTANLLWSFDIKNKTDTFEDATNSRPKPFEVDFFPRVPDLRRAIGKWAPAEVYKLFSHRYLLVAVIVASFQMVLQLWLEGRSLAIHRQGVDLLIEILETRNQPLHVSEPSSHGATSAGRQSRRSLQYQVLASQSTLVFTSPSLVRRGEEKNGYPALSPPAIPSPKPSTPPNPNSPSPPNPPPSQPPASSTKPNLAPSEPKPSSELPKPYQSPVHSPPPPNPQPPASAPAGGRDTQKTR  LENVQQDPSALNILQEANLQQGGNAGSIQTTDMAAPGAAIFVLDGNLINLSAKCVTALKQPMKFISRSHRTDIARIVFHVWLWGLTIWSLLLESIPHLAAVVISQYISAAFVSLDLKKSINLKEDFASVVNNDCDGVDVLPEFWGVLLKLDIVAATFASAIALTFTFLAFKLYSILDWRTFKKLGASRMVRIAHTLSLIFAAILQLNAYFVTVFLALWLREVLTYQWGTNSKNLFKTMGFKVTLGFFLALSAPWLLVGNFSLKHENLIGIIAFLIFDVALLTFTIFLLSQGFYRQMADLSAILNFTGIMACILMFASLLVAVACLLVFDRGLLAKVNRDSKSSSDTFERP  SMTDDEIAFPNNDVTPYNHQSNGFIFTSYDKNDNGFSDFATTSRTRTRLRNFGPLETLDHGIISKPSDEKSWISHNAQITRSVSGYSEYSASSSGLSYATTTFTPGRREAILMAWMSSRPSGSHPDGLDELQAVRSHPDGLDELQAIGIASQRPGSDYSNWFEYSNQKMAAAFFESNRIEFEYSNRFDSKCYALGSYLCMYM |
| PST_03397 | MGAKGWPKGPSYDSGITKGGLGGDSVLTIKTLPEHATRRRIWTKAFTPKAIVEYLPSIEIRLDEMISVIDDHVKQKKNVDLCTQLGCFVYNTLTGTDLLKNQEEKQRILTQMVRVVRQVGIVRNMPWLTPLVKAWPSTQRKEQHEFREFTRSMFLRRRNQGLGTQLDVFHYLLGEDTETGTRLTETELAADSTLLVITGSDTTRTVLLAFFLYILKHPGCMEQLQAELMAAPDLSPPTLSRLPYLNACLQETMRLQPPSPANLQRMCPPGGAVICGRHIPEGTKVRFSNYAIQRDERYFDDPDSFRPQRWLKNAEKEKMSEGEGPERFNEKAFFSFLIGPGACVAKSLAWMEMRLVVATLLTSYDVAFAEGFDPVAFESSWTDAYLLLIEEPFERSGQGFTEYLMFNGFTTFNWLLRIEHVFPEAKRPTV |
| PST_09715 | MTSRNRAIGTTKRKDPAFHEDPGWPLTNLYRAQRVGQLPTVIKNRARSLEEMTMRGLKYGPGHAITVPGVRMIDVSKPEWLEYIQKTNFDNYVKGPLFRNCMYDVFGDGIFVSDGQMWKRARHATSTIFTLKTFKTIIVPSSNKSMDELSQVLTSIAGRNHPIDFCDLFFRFTLDSFVHMTFGKDLGLLGIGYEDKGSNPFSVAFDYAQDQVDFRFVMPIGWRLIERLNIRSMGKRMKVSCGVLDDYAYSLIDERMSNLTSDPKKEKQATIHTDLLGLFMNARDERGGDLGRTELRDTTLNLIIAGRDTTAEVLSWSFFHLLMNEDLVLRIREEASGILGEEKRVTYENYKQFILAYAIVHETLRLHPSVPKDLKCVASDDQIPGGPRVEAGDMVRWSDWQMGRDASIWGPDCGEFKPDRWIDDEKGSIKQFGPYKFHAFNAGPRICLGMNLAIYQAVKVIVETFMSFELEFAPGWLENVPKSESIEGITSRYPTPMYRTSLTLPMDNPMMISVKKKKLPLVDM |
| **PST_09949**  **(*CYP51*)^a^** | MSSLLSPVFEFIGSFSLFVQFTIYLLLSIVSIVSINIFNQLIVPKNATTPPVVFHIFPIIGSAISYGIDPYAFLESCRKKYGNVFTFILINKKVTVALGLQGNALVLNGKLAQVNAEEAYTALTTPVFGTDVV**Y**DVPNAILMQQKKFIKSGLTTENFRKYVSTIVDETIGYIEDHIFQNPKTQQAVKDALKVASEITICTASATLQGPEVREGLNTSFADIYHDLDGGFTPLHFALPGLPLPSYRKRDLAQVAMRNFYLNIIKKRREDNREGQLGDMIDSLQGQTYKDGRPLTDKEIAHIMIALLMAGQHTSAATGSWLILHLASRPDLVKELRKEQIELFGKPGQTDENELDPLDLERVQSPLMIACIKEVLRLHPPIHSIMRKVKSPITVPRTLASLNEDTPYIIPSSHFVLAAPGTAQLDPSIWTAPDEFEPSRWLNQPSPFKTSTTTTSGEESTAQEEMVDYGFGMISSGANSPFLPFGAGRHRCIGEQFAYIQLSTIGATFIRNCDIELITDQFPKPDYTTMLVCPLKPRDIKFTRRNHL |
| PST_10075 | MLSSAALYSLLMGGFAYLAYLLFEYRDRAIGTTRRKDPLFRDVPGWPLIGQLVQSLTDMSQPLEASTIMALKLRPGLRITVPGVRIIDISKPEWLEYIQKTNFDNYIKGPMFRSLMLDLFGDGILVTDGAMWKRARMVTSRIFSANTFKTVIQPSVDQSVDGLLKVLQKTSDENGEVDFCNLFTRFTLDSFVKMTFGRALGLYADESSAEMESDAASHKKVSSPEDFADAFEFAQKQIDFRFTVMTGWELYEKLNSSVGGKMKRSCGTVHEYACALIDERLAKISSDDDFTNAETYANDFLGLMMAVHRQRGHSLNRLELRDAALDCQIRQESAELLGKYPAQQGRVTHENYKHFTCTYSALLETIRLHPPVPKNLKFAKAADVIPGGPTIEAGDCVTWSDWQMARDPEVWGPDCGEFKPERWIDETGKIQSFSNFKFHAFNGGPRQCLGMNMAIFVNIKAIVEMLQTFDLEFSEGWLENVPKCGEIEGITSSYPTPQYQPSLTLPMKHAMMISVKPRISQDPHE |
| PST_10079 | MNCQSISSIINFREPPTKLPTKLTDTYHDSYRHFFPATTVVIEQLAQLNERIHHSEKSLDGPCASKVILSSARPLTNTNEALHGLARDLPGSILDISRPWEADTIIGLKLRPGFSITLPGARVVEVSKPEWIEHIQKTNFENYVKGEMFQSLMADLFGKSILVTDGADWKRSRLVTSRVFHISVFKTVVEPRINPTMIEVLDVLQVSSDEGRAIDFSNLFNRFTLDLFVEMTYGKKLGLLGEESRIRNGDPAGSSYSEVFSDAFDFSQKYMDSRFHVAVIWQWIQSINFREKEQMKLSCHTIHGLAYTSIDEKMSKSAPEEDYISDEDESQEDFLGLIMGYHLQRGHALTRDELRDDSLSFVLAGRDATAQSLSWCFFHLLMNKDIVSRIRQEAADILGAESDNQASVTPENYRQFICTYASLLEAIRLHPGVPKNVKFAMADDKIPGGPTIEAGDCVTWSDWQLARDPEVWGPDCGQFVPDRWIDETGNIRHFGNFKFHSFNGGPRLCVGMNMAIYIAVKSIVDILINFDLEFAKGWLEKAPMSEEIPGIKTDYPTPQYRPSLTLPMKNPMMISATPRALYKNNKRQLKV |
| PST_10090 | MSDLSSHLKVLLLLLITVKFLTLLIKYRKRGVGTSKRQDVYDDLPGWPLLGLLPEVILNSRNLLEWAAQKTLVHGVGYSGSLFQEVMSDVFGNGIFVTDGAAWKTSRQTTARIFNTNNFNNIITPAVHKTLASFMDVLTFHCETQNPVEMDDLLHRFTLESFVKMTFSQDMGSLKAGLLLSEQTEQPFAESFDYVQKQLDLQFILTAIWIRLGRLVGNRPKMVAARRTLENYAYTLIDSRTANPNKDTEVYHDLLGLFMSFTDEKGLSMSRSELKDSALNLIIAGRDTTAQALSWTFFHLVRNPDVVEKMRIEIDKLTTSNDELVDYSNYKHFTYHLAVFYEALRLHPSVPKNAKFAVNHDKIPNGPLVQPGDFDWQMARDPSIWDQIAQNLNLRDG |
| PST_10499 | MQRSLTVSDILVLISIAILSWLGWFSRRYHDGKGKKIPQPAGLPIVGNLFQVRQPNPWIQMAKWTKEFGPIYRLKMGRSDLIVLGSPKIAVELLEQRSSKYSSRPRNIMTSEYVSKGLRLTFMPYNDLWRRQRKLLHLLTQPKAASAYQPIQSQESAQLCLDLLRFPNHHWNHFQRYAGSTVLQIAFNRRALSIRDPAITKMRECNSKMIETAVPGRYLIDSMPILRYLPQIISPWKRYGNQLFDQTLTLFSELYREVSEKLHLQPLDHSDSSPDACFVARIESLKESYQLSDDQAIFLAGAMFGAGSDTTADAIETFIFACAANPEKVANAQEELDRVIGRDRLPEFSDEDDLIYCGAMVRELLRWRTVIPGGLAHMTTEDDEYEGHFIPKGTTIVANHWSIHLDEKTYKDPEKFIPERFIDPQSGQLIGTKWSTYGHHAFGFGRRICPALHIANKSLFITFTRILWSFNIKIKESNLMSPEEFIKSIKFSTGFSSHPIDLNKSGSIEIIPRDPFSTLKTLVEAVENNGLDPIKLS |
| PST_11794 | MWRQAMCDMAFGGLFIESWNSACWGDDELQGPEDADYFNLHSSAYDFFALALTALAGTDLLKNQEEKQRILTQMVRVVRQVGIVRNMPWLTPLVKAWPSTQRKEQHEFREFTRSMFLRRRNQGLGTQLDVFHYLLGEDTETGTRLTETELAADSTLLVITGSDTTRTVLLAFFLYILKHPGCMEQLQAELMAAPDLSPPTLSRLPYLNACLQETMRLQPPSPANLQRMCPPGGAVICGRHIPEGTKVRFSNYAIQRDERYFDDPDSFRPQRWLKNAEKEKMSEGEGPERFNEKAFFSFLIGPGACVAKSLAWMEMRLVVATLLTSYDVAFAEGFDPVAFESSWTDAYLLLIEEPFEVTFTPKPGRLR |
| PST_14483 | ISAIGTSKRKESNFPEIPGWPILGQIPEIIMNFSRPLELGMARHLKFRPGWSVTMPGLRLIDISKPEWLEYVQKTNFHNYVKGPRFHSVMADVFGNGIFVTDGAQWKKSRHLLAPLFTIKTFKVCISPALRTNLDTLHESMQLASESRPVIDICDVLFKFSMNFLVYATLGKDMGQLDQLHLPSIQPSSPSADSKFVDAFEHAQKQLDLRFALLTSWKVVEKVNVSMGRKIASACQTLHDYASLLIDERLTSLGQGPQEYDETPTDLLGFFIKNRKEMGGDLDQAELKETFLNLIIAGRDSTAEALTWAFYHLLMNKDIVNKIREEASEVTGKDRSGQVTYENSKEFKWAHAVILEALRLHPSIPKNVRFALKADKIPGGPVIEAGDAVRWSDWAMARDPQVWGDDCLEFRPARWIDDNGNIKQFGHFKFHAFNGGPRVCLGMNFAILQCICMIVEVFHNFELELEPEWLAQVPKTPIVGHTSPNAYGTPKYKPSLTLPMAQSMMVTIKPY |
| PST_14792 | MSDLSSHLKVLLLLLITVKFLTLLIKYRKRGVGTSKRQDVYDDLPGWPLLGLLPEVILNSRNLLEWAAQKTLVHGVGYSVTMPGMRLIEITRPDWIEHVQKTNFQNYVKGSLFQEVMSDVFGNGIFVTDGAAWKTSRQTTARIFNTNNFNNIITPAVHKTLASFMDVLSFHCETHNPVEMDDLLHRFTLESFVKMTFSQDMGSLKAGLLLSEQTEQPFAESFDYVQKQLDLQFILTAIWIRLGRLVGHRPKMVAARRTLENYAYTLIDSRTANPNKDTEVYHDLLGLFMSFTDEKGLSMSRSELKDSALNLIIAGRDTTAQALSWTFFHLVRNPDVVEKMRIEIDKLTTSNDELVDYSNYKHFTYHLAVFYEALRLHPSVPKNAKFAVNHDKIPNGPLVQPGDFDWQMARDPSIWGPDCTEFKPSRWIDESGNLRQFGQWKFHAFNGGPRICIGMHLATLEAIACLVQVVRTFDLEFEPGWFENVPKIRKISPDSTEQTPRYASSLTLPMANPLRIVVRKRNSQ |
| PST_16901 | MTELIPATMRLIHNEIVQNFRVIFSLFICLLSPVILSTITRRRRCLPLPPGPKSYPFMRYSFGPYQWRQMEEITKEYGPVSSVKLGNNKLLVIVGRVEPALALLEGRSSIYCDRPQLEMAGNIMSGGLRTLCLPYGDRWKRFRRVLHSQLDTKAATSYQSIQERASRQMILDIVERPKDFTEALTRYAASVIIKITYGKLTPIHHGDAEVVKVIKTLSRFTRATRIGAHSVDRFPWLRHIPGWVAQGRKWHQEELDLFSSQVDGVRSEMNIPGKRDSCFTSYMLERQKEFSLSDNEAAYLAGSLFGAGSDTTSAALAVVIFAAASHPSEVKKVQDELESVVGNGRMPTFDDYPELPWVSAFVSETFRWRPVSAAGFMHATVKDDVYEGHFIPAGSWVIGNHWSIHRDESVFPEPEKFDLTRWLTIDEESGKTVLRPDMRHFAYGFGRRRCAGVTIADRSMFINTANLLWSFDIKNKTDTFEDATNSRPKPFEVDFFPRVPDLRRAIEEMGAC |
| PST_20448 | MSQLALLLSYLGPLVLSKFLVESFSIHPTFLDRLVVFCLSWPIAREIRLWLQQRELRARASSRGAVLAPLALSRLPFGLSVLLTRLRMIHSGSPGDVMDLFKTGVAHPANSDQHTKVFRSRVMGVETLWTLDHDDAKYLLSTGFGNFGKSPFFKAGFQRLLGDGVFASDQRGLWAWHRSLTRPHFVRERIADVVAMEEHSQRVATWLSTQTDLGKSVDIQDIFARYTLTVGTQHLFGRCVDSLNDLLHDRIQNGPNPADFAQNFVAAQHWAIINSLLPPLLISLGFRLKDKATEDVRQVVDTLIQDASLSLASDIKSNASDTDKVEGEIASENLLDHLLTSGCSKELVRHECLNILLAARDTTASLLSSCFYEIARDSPRKTEIWRKLKEEVERLGSGIDGLLTLDQVREMKYLRAVLNESLRLHPPVWANTRHAFEDDVLPSGVFVPAGTDCRFFIREFQRNPAVWGQDAEEFDPDRWIDSRKVLQIKDPLCFQPFSAGPRICLGQQFALTEASIMIIRIVEQFEGVDLDLSEGPVGAEAPAVVLTFRGGLKVRFKR |
| PST_23841 | MAIADCPPVAGYTPTPQNNSSGYQNDGANPSNVETNPRPDSTKFSCNLLVTHLSPVACDPVDDHSPRTSSPSSPWTQRISILWVFIRPIVSRPCTHHPSIVQHAFWPAHRHFESSHWRQMEQITEKYGPVSSVRLGNKLLVIVGRVEPALALLEGRSSIYCDRPQLEMAGNIMSGGLRTLCLPYGERWKRFRRVLHSQLDTKAAISYQPIQERASRQLILDIVERPKCFTEALTRYAASVIIKITYGKLTPIFHDDAEVLQVIKTLNRFTKAARIGSHAVDRFPWLRYVPGWVAQGRKWHSEELDLFSSQVDGVRREMNIPGKRDNCFTSYMLERQKEFSLSDNEAAYLAGSLFGAGSDTTAAALAVVIFAAACHPSEVKKVQDELESVVGNGRMPTFDDHLELPLVSAFVCETFRWRPVSAAGFMHAAVKDDVYEGHFIPAGSLVIGNHWSIHRDESVFPEPEKFDLTRWLMIDEESGKTVLRSDMRHFAYGFGRRKCAGVTIADRSMFINTANLLWSFDIKNKMGKDGNLIELDTMAFEDATNSRPKPFEVDFVPRVPDLRRAIEEMSAC |
| PST_24116 | MKLLNVVILSMVILIQGTFVRADFKCLDKSKPVGYCTLDGAPVPVTPGKPYTMTKAPQSDDKKGFHCSGRVLESCCTKKFKPENPLSKVTYGSSCKTVGNFALDIDDTETMMMVISLILTILEYSIIISLAGISYYYILGYSWNKHQINHFPGPFLAKFSRLWLGYHTRFGKRYQIIHELHQKHGRFVRIAPNELSIADPDAIAVVLGHGTGTTKSKFYDAFVAIHRGLFNTRDRADHTRKRKIISSTFSQKSILEFEPYIADTLACFLRKMDQVAAEPNSVHDHKPQSDSKEDDDEGWRILDILPWFNYLAFDIIGDLAFGERFGMIERGADIAGVEKEGEVLYLPAIQILNERGEFSATQGSLPAGLRPYMKYIDPWFSRGAASVKNLTGIATNQVNLRLSQTGQSRKDLLARLQTGQDADGNPMGKDELIAEALTQLIAGSDTTSNSSCMILWWAIKHPNVYQKLMQELDENLGTEAGVVSYADSKDLKYLNACINETLRIHSTSSIGLPRILPRTVSFKGHILSKGLVCSVPTFTIHHDAEVWGDPFTFRPERWLEPNAKDREKAFEPFSCGPRSCIGRNLAMMELYMITATILKRYEFALPDPNLAELETREGFLRKPLNCWVKFKLRSD |
| PST_24224 | MISTVHAMGSAELPFLSLHLLSHSFSGIFSTHPFDFGNLLLREHSTTNSWIQKNAVLVLRRWTEWLTGPHGFYIISFLISGLLIYDAFRPRPLNGIPTSGHYSWAIGDSGALYRHMKVTGTRTTFFTDQANRVGPLSQVMLGPAGSWFGKLTGLGSHIIVLADGQQLVEVCAKRSREFPDPGLTLDIYKGIIPNGQLALPTGHAFKHHRRAIAHSMSSSHLSQVTPKITNSVIELIQLWKKRSQILDESGETYFKAAQDLRLSTMDTISDIIFGKPFGVMSARLSHLETTNGTLSADSRPQFPTLARALNVIVREVAMCYVAPSKSLFWFYQRLFNWEWLRAKSTVFSYLKKEVEKERIALAEEQSFGGVDSKDPDNVDSVLSLLVKDEQQSKLRGEKPLSTDEITQELLVYWVAGHGTMACTLAWAVKLLSNNPEVQYRLRDELVNTLPSLNERSPTFSDLKVCSSDLSYLDATCFEILRYGKVLGEVSRTSRVDAVVMGHLIPKDTVVMMPHSRLGGSQDDDSEFRPERWIDCDGKFDAQLPGPLHVFGHGQRGCFGKNLAVLELKLYIAMLCMELFFDRVPDHVNSMDATELVANHPLTCVIRPIPWSKINNNL |
| PST_24379 | MSSLIGKWVLGSLAYLLYLLIKYRNRAIGTSKRDDPPMTDIPGWPLFAFFYLLIVSMECNILILASLLLLDARLGQLPRIIANRSRSLEDTTSTALKFGPGYSFTVPGLRIIDVSKPEWIEYLQKTNFNNYVKGPLLQPVMLDVLGHGIFVADGPAWKRARQATVSIFTHKTFKTIILPSVNRSMIGFAQVLTTAAEQGLTIDICDLFFRFTLDSFVRMTFSKDLGICDAGYLSHSGLSTALPQLTDPTTEFPKAFDLAQNQMDFRLTATPGWQLLEWLNIGSMGKRMKESCQILDEFVYSLIDQRLADLSRKPDLHDKESSHPDLLSLFITTRDERGGGLGRAELRDTALNLIIAGRDTTAQALSWAFFHLVMNKELICKIRDETIEVLGDDPVDDQLSVSYENYKQFVWTHAVVLEALRLHPSVPKNAKIALSDDKIPGGPTIEAGDMVRWSDWQMARDPSIWGDDCGDFKPQRWIDDTGSIKQFGQFKFHAFNGGPRLCPGMNLAILEAVKVIVQVLREFELEFAEGWLENVPKGEFIEGVTSQYRTPMYSASLTLPMDNPMMISVRLRQLD |

^a^ The *CYP51* gene was used in this study for developing KASP markers to characterization the nucleotides at the 401 position. The change from nucleotide A to T will change the amino acid at position 134 from tyrosine (**Y**, highlighted in bold), to phenylalanine (**F**).
